# Quantification of hyperpolarisation efficiency in SABRE and SABRE-Relay enhanced NMR spectroscopy†
†Electronic supplementary information (ESI) available. See DOI: 10.1039/c8cp05473h


**DOI:** 10.1039/c8cp05473h

**Published:** 2018-10-10

**Authors:** Peter M. Richardson, Richard O. John, Andrew J. Parrott, Peter J. Rayner, Wissam Iali, Alison Nordon, Meghan E. Halse, Simon B. Duckett

**Affiliations:** a Centre for Hyperpolarisation in Magnetic Resonance , Department of Chemistry , University of York , UK . Email: meghan.halse@york.ac.uk ; Email: simon.duckett@york.ac.uk; b WestCHEM , Department of Pure and Applied Chemistry and CPACT , University of Strathclyde , Glasgow , UK

## Abstract

*para*-Hydrogen (*p*-H_2_) induced polarisation (PHIP) is an increasingly popular method for sensitivity enhancement in NMR spectroscopy. Its growing popularity is due in part to the introduction of the signal amplification by reversible exchange (SABRE) method that generates renewable hyperpolarisation in target analytes in seconds. A key benefit of PHIP and SABRE is that *p*-H_2_ can be relatively easily and cheaply produced, with costs increasing with the desired level of *p*-H_2_ purity. In this work, the efficiency of the SABRE polarisation transfer is explored by measuring the level of analyte hyperpolarisation as a function of the level of *p*-H_2_ enrichment. A linear relationship was found between *p*-H_2_ enrichment and analyte ^1^H hyperpolarisation for a range of molecules, polarisation transfer catalysts, NMR detection fields and for both the SABRE and SABRE-Relay transfer mechanisms over the range 29–99% *p*-H_2_ purity. The gradient of these linear relationships were related to a simple theoretical model to define an overall efficiency parameter, *E*, that quantifies the net fraction of the available *p*-H_2_ polarisation that is transferred to the target analyte. We find that the efficiency of SABRE is independent of the NMR detection field and exceeds *E* = 20% for methyl-4,6-*d*_2_-nicotinate when using a previously optimised catalyst system. For the SABRE-Relay transfer mechanism, efficiencies of up to *E* = 1% were found for ^1^H polarisation of 1-propanol, when ammonia was used as the polarisation carrier.

## Abstract 2

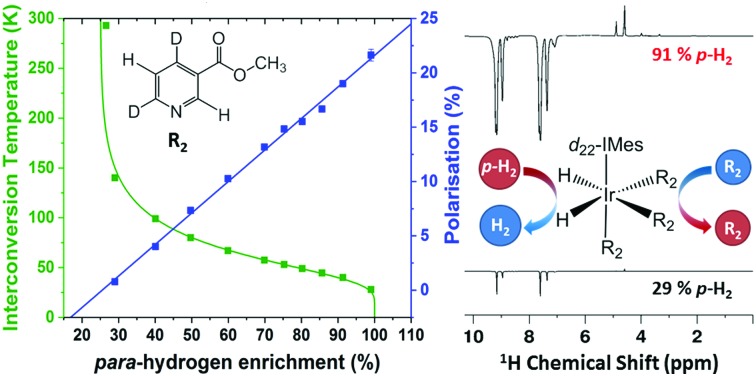
The linear relationship between the level of *p*-H_2_ enrichment and the polarisation of the target molecule provides a route to quantifying the efficiency of the signal amplification by reversible exchange (SABRE) and SABRE-Relay NMR hyperpolarisation methods.

## Introduction

Nuclear magnetic resonance (NMR) spectroscopy is a widely used analytical tool for the identification, characterisation and quantification of molecules. However, many applications of NMR spectroscopy are limited by the relatively low sensitivity of the technique. The signal in an NMR experiment is derived from the population difference across nuclear spin states, the so-called polarisation. At thermal equilibrium, the nuclear polarisation is dictated by the Boltzmann distribution and is proportional to the applied static magnetic field.^1^

The quest for increased polarisation and hence improved sensitivity has led to the development of large and expensive NMR spectrometers that use superconducting magnets to achieve very strong and homogeneous magnetic fields. An alternative route to increased NMR sensitivity is through hyperpolarisation. Hyperpolarisation refers to any method that generates a nuclear polarisation that is significantly larger than that dictated by the Boltzmann distribution at thermal equilibrium.^2^–^5^ A wide range of hyperpolarisation methods have been developed, each with different advantages and challenges. The most widely used methods today include dynamic nuclear polarisation (DNP),^6^,^7^ dissolution DNP (D-DNP),^8^–^10^ spin exchange optical pumping (SEOP),^11^,^12^ brute force hyperpolarisation,^13^ and *para*-hydrogen (*p*-H_2_) induced hyperpolarisation (PHIP).^14^–^16^

In this work we focus on PHIP methods, which use the singlet nuclear spin isomer of molecular hydrogen, *p*-H_2_, as the source of polarisation. In PASADENA and ALTADENA, the original PHIP experiments of Bowers and Weitekamp,^17^,^18^ hyperpolarisation is achieved by using *p*-H_2_ in a hydrogenation reaction. PHIP has been widely used for the mechanistic study of hydrogenation reactions^19^–^24^ and the generation of hyperpolarised MRI contrast agents for clinical diagnosis.^25^–^27^ More recently, the range of potential applications for PHIP has been increased with the introduction of the signal amplification by reversible exchange (SABRE) method.^28^–^30^ SABRE is a non-hydrogenative version of PHIP that catalytically transfers spin order from *p*-H_2_ to a molecule of interest without chemical alteration of this target molecule.

There are many benefits of the SABRE approach when compared to other hyperpolarisation techniques. Firstly, the hyperpolarisation transfer step occurs outside of the NMR spectrometer in a weak polarisation transfer field (PTF) of the order of 0–10 mT.^30^–^33^ Therefore the observed hyperpolarisation level is independent of the strength of the detection field. Secondly, polarisation is generated rapidly, over just a few seconds, thereby allowing for quick and simple experimental implementation. Thirdly, as no chemical change occurs to the target analyte, the process is fully reversible. Hence, hyperpolarisation can be re-established easily by supplying fresh *p*-H_2_.^34^ Finally, *p*-H_2_ can be generated relatively easily and can be stored for weeks to months.^35^–^37^ One of the key limitations of SABRE is the range of molecules that are amenable to hyperpolarisation due to the need for the target analyte to reversibly bind to the SABRE catalyst on a suitable timescale. This issue has been addressed recently by the introduction of a new mechanism for polarisation transfer called SABRE-Relay.^38^,^39^ In principle this extends the SABRE method to include target molecules with any functional group that contains an exchangeable proton. Examples include, but are not limited to, alcohols, carboxylic acids, amines, amides, carbonates, and phosphates.^38^

In this work we explore an aspect of SABRE that has implications both for the reproducibility of SABRE enhancement levels and the cost of the technology for a given application. Specifically, we study the relationship between the level of *p*-H_2_ enrichment of the H_2_ gas used in the SABRE reversible exchange reaction and the resultant level of hyperpolarisation on the target analyte. This relationship has cost implications because the level of *p*-H_2_ enrichment is determined by the temperature at which the *ortho*-to-*para* conversion is achieved.^36^,^40^,^41^ For example, cooling H_2_ to 77 K *via* liquid N_2_ is very inexpensive but only results in ∼50% *p*-H_2_ enrichment, whereas conversion temperatures below 30 K are required to reach >95% *p*-H_2_ enrichment.^35^ Instrumentation and maintenance costs in this temperature regime can be much higher.

Herein we experimentally determine the dependence of SABRE-derived hyperpolarisation on the *p*-H_2_ enrichment level for different substrates, catalysts, NMR detection fields, and for both the standard SABRE and SABRE-Relay polarisation transfer mechanisms. By relating these empirical results to a simple model for *p*-H_2_-derived polarisation, we extract an efficiency parameter that describes the fraction of *p*-H_2_-derived polarisation that has been transferred to the target analyte. This measure can be used to evaluate the efficiency of a given experimental implementation of SABRE, independent of the *p*-H_2_ enrichment level, and can also be used to determine the required level of *p*-H_2_ enrichment for a given SABRE application.

## Theory

### Polarisation of H_2_ as a function of *para*-hydrogen enrichment level

**Fig. 1:**
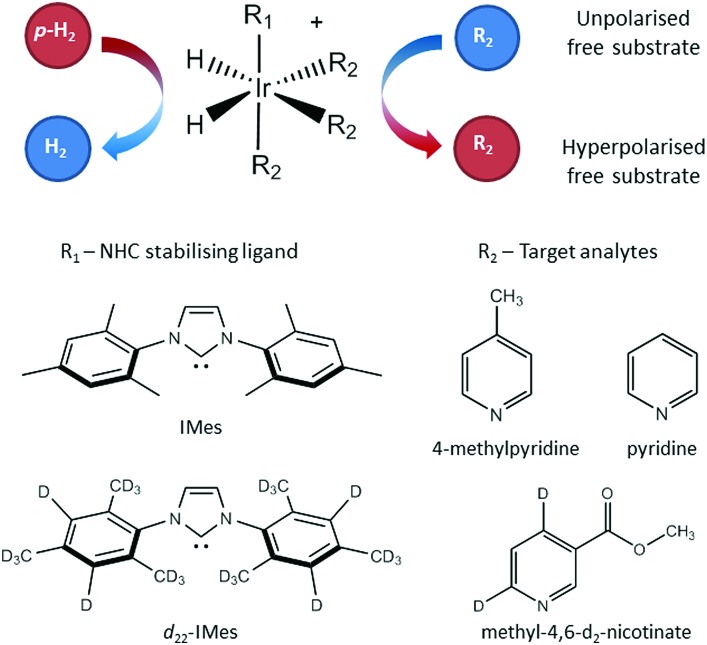
In the SABRE hyperpolarisation method, the active catalyst reversibly binds *p*-H_2_ and one or more molecules of the target analyte (R_2_). This establishes a *J*-coupling network between the *p*-H_2_-derived hydrides and the NMR-active nuclei in R_2_ such that polarisation transfer can occur. Rapid chemical exchange of both *p*-H_2_ and R_2_ leads to the build-up of hyperpolarised R_2_ in free solution. This process is reversible, meaning that hyperpolarisation can be regenerated upon supply of fresh *p*-H_2_. The form of the SABRE catalyst can be changed to optimise SABRE efficiency for different analytes by varying R_1_, a stabilising N-heterocyclic carbene.

The SABRE technique, illustrated schematically in Fig. 1, is a catalytic process for transferring the nuclear spin order of *p*-H_2_ to a target analyte.^30^ The polarisation transfer process is mediated by a transition metal complex that reversibly binds *p*-H_2_ and one or more molecules of the target analyte (R_2_ in Fig. 1). The active SABRE complex establishes a *J*-coupling network between the *p*-H_2_-derived ^1^H nuclei and the NMR-active nuclei in the bound target molecule. With appropriate coupling constants and polarisation transfer fields (PTF, typically 0–10 mT), there is a spontaneous transfer of spin order from *p*-H_2_ to the target molecule.^30^,^31^,^33^,^42^,^43^ As *p*-H_2_ and the target molecules undergo rapid chemical exchange between the bound and free forms, there is a build-up of hyperpolarised target molecules in free solution. Under a continuous supply of fresh *p*-H_2_, the level of polarisation of the target molecules in solution will reach a steady-state that is dictated by the efficiency of the polarisation transfer process, the rate of ligand exchange, and NMR relaxation. This steady-state is typically reached on a timescale of tens of seconds.

**Fig. 2:**
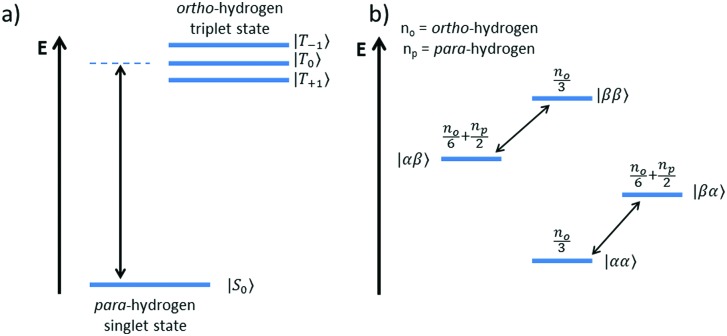
Energy level diagrams for the nuclear spin states of H_2_ (a) before and (b) after undergoing a pair-wise hydrogenation reaction that leads to chemical or magnetic inequivalence between the two nuclei. Note: the two diagrams are not drawn to scale. The relationships between the states in (a) and (b) are as follows: 
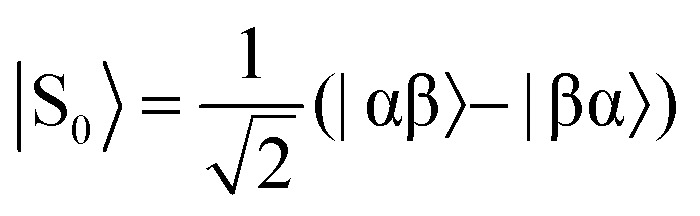
, 
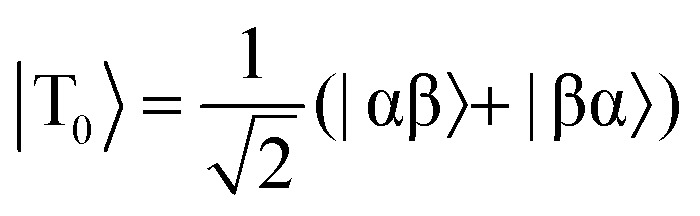
, |T_+1_〉 = |αα〉, and |T = |αα〉 = |αα〉, and |T, and |T_–1_〉 = |ββ〉. = |ββ〉 = |ββ〉..

The overall efficiency of SABRE can be quantified by the proportion of the available polarisation (derived from *p*-H_2_) that is transferred and subsequently detected on the target analyte in free solution. Molecular hydrogen has two nuclear spin isomers: *p*-H_2_, a nuclear singlet state, and *ortho*-hydrogen (*o*-H_2_), a nuclear triplet state. The energy level diagram for H_2_ is given in Fig. 2a, where α and β denote the spin-up and spin-down states of the protons in H_2_, respectively, and the singlet and triplet states are defined as: 
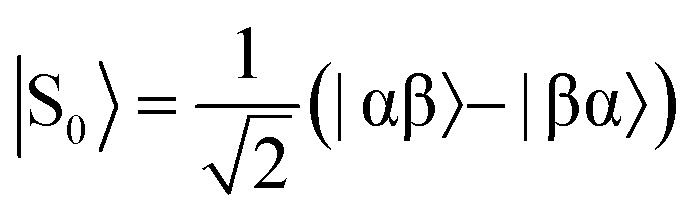
, 
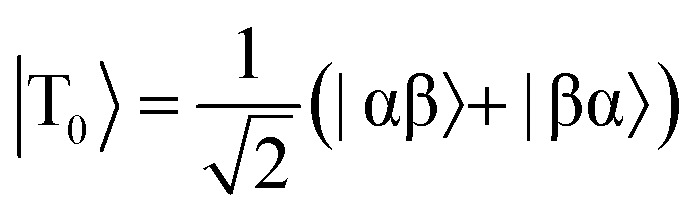
, |T_1_〉 = |αα〉, and |T = |αα〉 = |αα〉, and |T, and |T_–1_〉 = |ββ〉. At room temperature, all four energy levels are roughly equally populated and so H = |ββ〉 = |ββ〉. At room temperature, all four energy levels are roughly equally populated and so H. At room temperature, all four energy levels are roughly equally populated and so H_2_ contains approximately 25% *p*-H_2_ and 75% *o*-H_2_. Following a chemical reaction, where the protons from H_2_ are transferred into chemically and/or magnetically different environments in the product molecule, the energy level diagram for the H_2_-derived protons can be drawn as shown in Fig. 2b, where the exact separation of these energy levels will depend on the strength of the applied magnetic field and the chemical shift and *J*-coupling network of the H_2_-derived protons within the product molecule.

Consider a hydrogen addition reaction involving H_2_, where the fraction of molecules that are in the *para* and *ortho* forms are defined as *n*_*p*_ and *n*_*o*_, respectively. If we assume that the population associated with the *ortho* form is distributed evenly amongst the three triplet states, we can assign a population of *n*_*o*_/3 to each of the triplet states and a population of *n*_*p*_ to the singlet state. This would be the case at thermal equilibrium in a very weak magnetic field. Following a pair-wise hydrogen addition reaction, this population distribution will be mapped onto the energy levels in Fig. 2b such that the populations of the superposition states, 
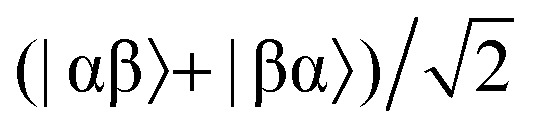
 and 
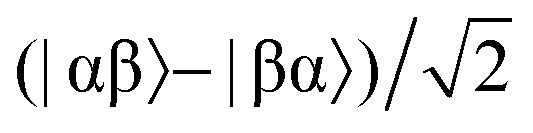
, in H_2_ are divided evenly between the |αβ are divided evenly between the |αβ〉 and |βα〉 states of the protons in the product molecule. and |βα are divided evenly between the |αβ〉 and |βα〉 states of the protons in the product molecule. states of the protons in the product molecule.^35^ As a result, the |αα As a result, the |αα〉 and |ββ〉 states of the product molecule will have populations of ( and |ββ As a result, the |αα〉 and |ββ〉 states of the product molecule will have populations of ( states of the product molecule will have populations of (*n*_*o*_/3), while the |αβ/3), while the |αβ〉 and |βα〉 states will have populations of ( and |βα/3), while the |αβ〉 and |βα〉 states will have populations of ( states will have populations of (*n*_*o*_/6 + *n*_*p*_/2). We define the latent polarisation of the system as the sum of the population difference between states that undergo single-quantum transitions, *i.e.* between |βα between |βα〉 (or |αβ〉) and |ββ〉 and |αα〉. The polarisation of the pair of protons from H (or |αβ between |βα〉 (or |αβ〉) and |ββ〉 and |αα〉. The polarisation of the pair of protons from H) and |ββ between |βα〉 (or |αβ〉) and |ββ〉 and |αα〉. The polarisation of the pair of protons from H and |αα between |βα〉 (or |αβ〉) and |ββ〉 and |αα〉. The polarisation of the pair of protons from H. The polarisation of the pair of protons from H_2_ in the product molecule, *P*_H_2__ can therefore be defined by eqn (1), where we have simplified the relationship using the substitution: *n*_*o*_ = 1 – *n*_*p*_ and the population difference between each pair of energy levels is doubled to account for the two possible transitions.1
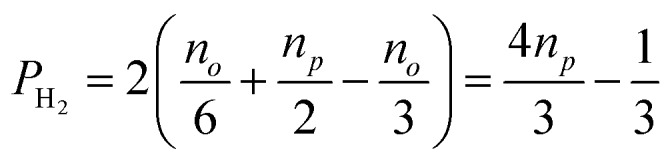
We note that at the moment of pair-wise *p*-H_2_ addition, there is no detectable magnetisation because there is an even balance between the Δ*m* = +1 and Δ*m* = –1 transitions. However, in the case where the symmetry of H_2_ is broken in the product molecule, eqn (1) can be used to define the latent polarisation that will become observable as a result of coherent evolution under differences in chemical shift or *J*-coupling between the H_2_-derived protons.^31^,^35^

Inspection of eqn (1) reveals that when H_2_ is at equilibrium at room temperature where the relative populations of the two isomers approach *n*_*p*_ = 0.25 and *n*_*o*_ = 0.75, the polarisation approaches zero, as expected. However, in the limit where *p*-H_2_ enrichment, *n*_*p*_, approaches 1, the polarisation also tends to 1, indicating the potential for large NMR signal enhancements. Therefore eqn (1) provides a conversion between *p*-H_2_ enrichment level, *n*_*p*_, and the latent polarisation of the pair of protons in H_2_, which can be unlocked by a chemical reaction.

### Quantifying *para*-hydrogen enrichment level

Transitions between the *ortho* and *para* states of H_2_ are symmetry-forbidden. Therefore, conversion will only take place in the presence of a catalyst, for example a paramagnetic species such as iron(iii) oxide or activated charcoal. To produce H_2_ gas enriched in the *p*-H_2_ state, it is cooled to the desired conversion temperature, *T*, in the presence of the catalyst. After conversion between the *ortho* and *para* states is achieved, the gas is heated up in the absence of the catalyst for use at room temperature. If carefully isolated from contact with paramagnetic species, the *p*-H_2_ enriched gas can be stored at room temperature for long periods of time (*i.e.* weeks to months).^36^,^40^,^41^

The level of *p*-H_2_ enrichment achieved by this process will depend on the conversion temperature at which thermal equilibrium is established. The absolute populations of the *p*-H_2_ (*N*_*p*_) and *o*-H_2_ (*N*_*o*_) states at thermal equilibrium are governed by Boltzmann statistics. Due to the coupling of the even rotational states to the singlet nuclear spin state of *p*-H_2_ and the coupling of the odd rotation states to the triplet nuclear spin states of *o*-H_2_, *N*_*p*_ and *N*_*o*_ can be given by eqn (2) and (3), where the rotational constant, *θ*_R_, is defined in eqn (4), *h* is Planck's constant, *k*_B_ is Boltzmann's constant, and *I* is the moment of inertia.^35^2
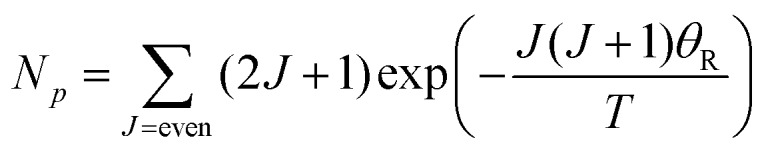

3
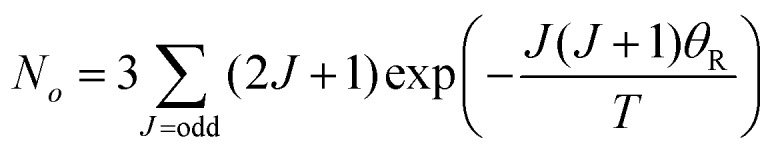

4
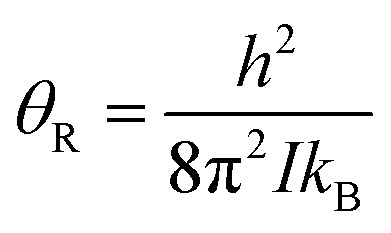
In an NMR experiment, the observed signal from H_2_ corresponds exclusively to *o*-H_2_ because *p*-H_2_ is a singlet and has no net angular momentum. Therefore, the amplitude of the observed signal for a fixed concentration of H_2_ will depend on the relative level of *o*-H_2_. If we define the observed NMR signal from *o*-H_2_ as *S*_*ortho*_ and the maximum observable NMR signal, *S*_max_, as that which would be observed in the case of 100% *o*-H_2_ enrichment, we can relate the observed signal (*S*_*ortho*_) to the populations of the two spin isomers, *N*_*p*_ and *N*_*o*_ as in eqn (5).5
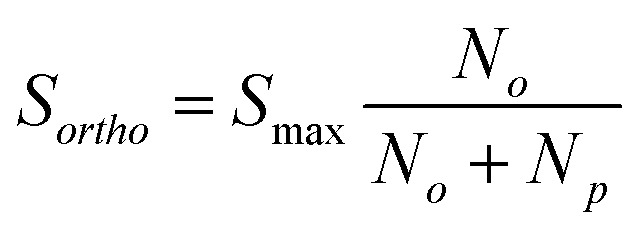
By inserting the expressions from eqn (2) and (3) into eqn (5), we can determine *S*_max_ and *θ*_R_ by quantifying the ^1^H NMR signal response for H_2_ gas that has been enriched at a range of conversion temperatures. Subsequently, the level of *p*-H_2_ enrichment, *n*_*p*_, can be calculated from either the NMR signal intensity for *o*-H_2_ (*S*_*ortho*_) or the conversion temperature (*T*) using eqn (6) and the definitions for *N*_*o*_ and *N*_*p*_ in eqn (2) and (3).6
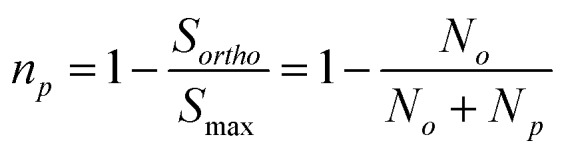



## Experimental

### *para*-Hydrogen production

The generation of *p*-H_2_ was achieved by using a closed helium refrigeration system which allows for temperatures down to 7 K. The system has been described previously but has had several modifications here.^44^ It consists of a compressor and low-temperature cooler, which provides a two-stage-closed-cycle helium recirculation pump. In order to get good contact with the H_2_ gas a copper block is used which is connected to the cooler. The copper block contains a tortuous void which introduces a significant residence time for H_2_ in the presence of the interconversion catalyst (activated charcoal). The gas is passed through the copper block from an inlet at the bottom of the copper block to an outlet at the top. Since the cold head reaches a temperature of 7 K and at this temperature H_2_ is a solid, the system has a feedback loop connected to a heater that is used to regulate the temperature of the copper block. A range of conversion temperatures was achieved by varying the heater power to stabilise at a given temperature. To hold the temperature at 140 K, the heater required around 60% of the maximum power, which was deemed to be the maximum safe operating level of the heater. Therefore, temperatures over the range of 140 K to 28 K were used. An additional point was acquired at 293 K by using thermally polarised H_2_ gas taken directly from the H_2_ cylinder. The feedback controller was able to hold the temperature of the cold head accurately to ±0.1 K, as measured by a thermocouple in the copper block. NMR tubes fitted with Young's valves can be attached to the apparatus *via* an adaptor. The system also includes a vacuum pump (Edwards RV pump), which can be combined with a H_2_ bypass line to purge/flush the lines with H_2_ before the addition of a sample. This vacuum pump also allows the removal of used H_2_ from the headspace of the NMR tube before the subsequent addition of fresh *p*-H_2_ gas. The hydrogen inlet was supplied with CP-grade hydrogen from an external cylinder *via* a regulator set to 3 bar (gauge) and the outlet pressure was monitored with a pressure gauge to ensure a fixed pressure of 4.0 ± 0.1 bar of gas was supplied at all times, measured with a digital pressure gauge (Baratron Pressure Transducer and Manometer MKS). When changing the temperature of the copper block, the system (including the void space within the copper block) was purged ten times before subsequent experiments were carried out to avoid any contamination from *para*-enriched H_2_ generated at a different temperature.

### Measuring *p*-H_2_ concentration with NMR

In order to determine the *p*-H_2_ concentration using liquid-state NMR, 0.6 mL of toluene-*d*_8_ was placed inside an NMR tube fitted with a Young's valve (GPE scientific). The deuterated solvent was degassed using a 3-stage freeze–pump–thaw procedure with an acetone and dry-ice bath. 4 Bar (absolute) of H_2_ gas was added to the headspace and the NMR tube was then shaken vigorously for 5 seconds to promote dissolution of the H_2_ into the solvent. Each measurement was carried out using a fresh sample in a separate NMR tube. ^1^H NMR measurements were carried out on a 500 MHz Bruker Avance III HD NMR spectrometer with a BBI probe using a single scan. This was repeated three times for each conversion temperature in order to take an average integral of the *ortho*-hydrogen signal. These average integrals were subsequently fitted to eqn (5) to obtain fitting parameters that were used in the determination of the *p*-H_2_ concentration (eqn (6)).

### SABRE and SABRE-Relay experiments

The SABRE enhanced ^1^H NMR response was detected on either a 400 MHz Bruker Avance III using a BBI probe or a 43 MHz (1 T) Magritek Spinsolve Carbon benchtop NMR spectrometer. The SABRE samples contained 26 mM of the target analyte and 5.2 mM of the SABRE pre-catalyst in the form [IrCl(COD)(NHC)] (where COD = 1,5 cyclooctadiene) where the N-heterocyclic carbene (NHC) was either 1,3-bis(2,4,6-trimethylphenyl)-imidazol-2-ylidine (IMes) or 1,3-bis(2,4,6-tris(methyl-*d*_3_)-4,5-*d*_2_-phenyl)-imidazol-2-ylidine (*d*_22_-IMes) (see Fig. 1). The target analytes were: 4-methylpyridine, pyridine and methyl-4,6-*d*_2_-nicotinate and the solvent was methanol-*d*_4_ (Fig. 1). Both of the catalysts and the methyl-4,6-*d*_2_-nicotinate were synthesised in-house,^45^ the analytes 4-methylpyridine and pyridine were purchased from Sigma-Aldrich. For each sample, a 7 mL bulk solution containing catalyst, analyte and solvent was prepared and 0.6 mL was subsequently distributed into ten different NMR tubes, each fitted with a Young's valve and degassed using a 3-stage freeze–pump–thaw-method in a bath of dry ice and acetone. The SABRE catalyst was activated by adding 4 bar *p*-H_2_ to the headspace of the NMR tube and shaking vigorously for ten seconds. This was repeated six times and then the sample was left inside the NMR spectrometer for a further ten minutes to ensure full activation of each sample. Once activated, a single-scan thermal ^1^H NMR spectrum was acquired as a reference for the SABRE enhancement factor calculation. For each subsequent SABRE experiment, the head-space of the NMR tube was evacuated and then charged with 4 bar *p*-H_2_ at the desired enrichment level and shaken for 10 seconds in a handheld Halbach array with a static field of 63 G before being manually transferred to the NMR spectrometer for detection.^46^ The sample transfer time was 3.5 ± 0.5 s and each measurement was repeated 5 times.

For the SABRE-Relay method, samples were made by first preparing a 7 mL bulk solution containing 5.2 mM of the pre-catalyst [IrCl(COD)(IMes)] in dichloromethane-*d*_2_ (DCM-*d*_2_) in a 10 mm diameter NMR tube fitted with a Young's valve. The sample was degassed using a 3-stage freeze–thaw–pump process using liquid nitrogen. Ammonia gas was introduced to the head-space of the NMR tube and dipped in liquid nitrogen quickly to promote condensation, and the tube was sealed and subsequently shaken vigorously for 10 seconds to promote dissolution of the ammonia. The amount of ammonia in solution was quantified using liquid state ^1^H NMR to be 42 ± 2 mM (see ESI† for full details). 26 mM (13.6 μL) of 1-propanol was added to the NMR tube and the solution was de-gassed using a 3-stage freeze–pump–thaw method with liquid nitrogen. The 7 mL bulk solution was distributed into ten different NMR tubes for analysis (each 0.6 mL). The SABRE catalyst was activated by adding 4 bar of H_2_ to the headspace of the NMR tube, which was then shaken for ten seconds and left overnight.

In order to calculate the enhancement factor and subsequently the polarisation, a single scan non-hyperpolarised ^1^H NMR spectrum was acquired with the same settings as the subsequent SABRE-enhanced detection. The enhancement factors, *ε*, were then determined by taking a ratio of the thermal and hyperpolarised integrals as in eqn (7).7
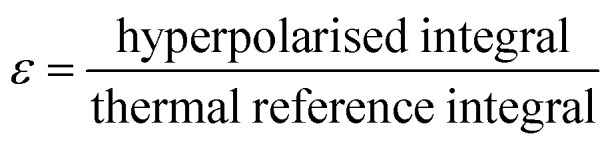
Polarisation, *P*, was calculated by scaling the enhancement factor to the thermal polarisation level in the detection field using eqn (8), where *γ* is the gyromagnetic ratio, *B*_0_ is the detection field, *T* is the sample temperature, *ħ* is the reduced Planck's constant, and *k*_B_ is Boltzmann's constant.8
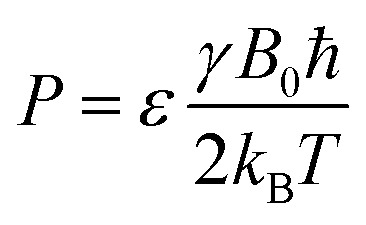



## Results and discussion

### *para*-Hydrogen enrichment calibration

Quantification of *p*-H_2_ enrichment has been demonstrated previously using a range of experimental methods including thermal conductance,^47^ Raman spectroscopy,^48^,^49^ and ^1^H NMR spectroscopy, where the NMR response of *o*-H_2_ is measured directly in the gas phase.^40^ Here we took a liquid-state NMR approach in which the *para*-enriched H_2_ is dissolved in a fixed volume of toluene-*d*_8_ and the ^1^H NMR response for *o*-H_2_ in solution is quantified. A similar approach has been employed previously to study the effect of *p*-H_2_ conversion rates in diamagnetic solvents.^41^ The main advantage of liquid-state NMR is the increased sensitivity over the gas-phase approach, particularly when quantifying high levels of *p*-H_2_ enrichment where the residual signals from *o*-H_2_ are very weak.

**Fig. 3:**
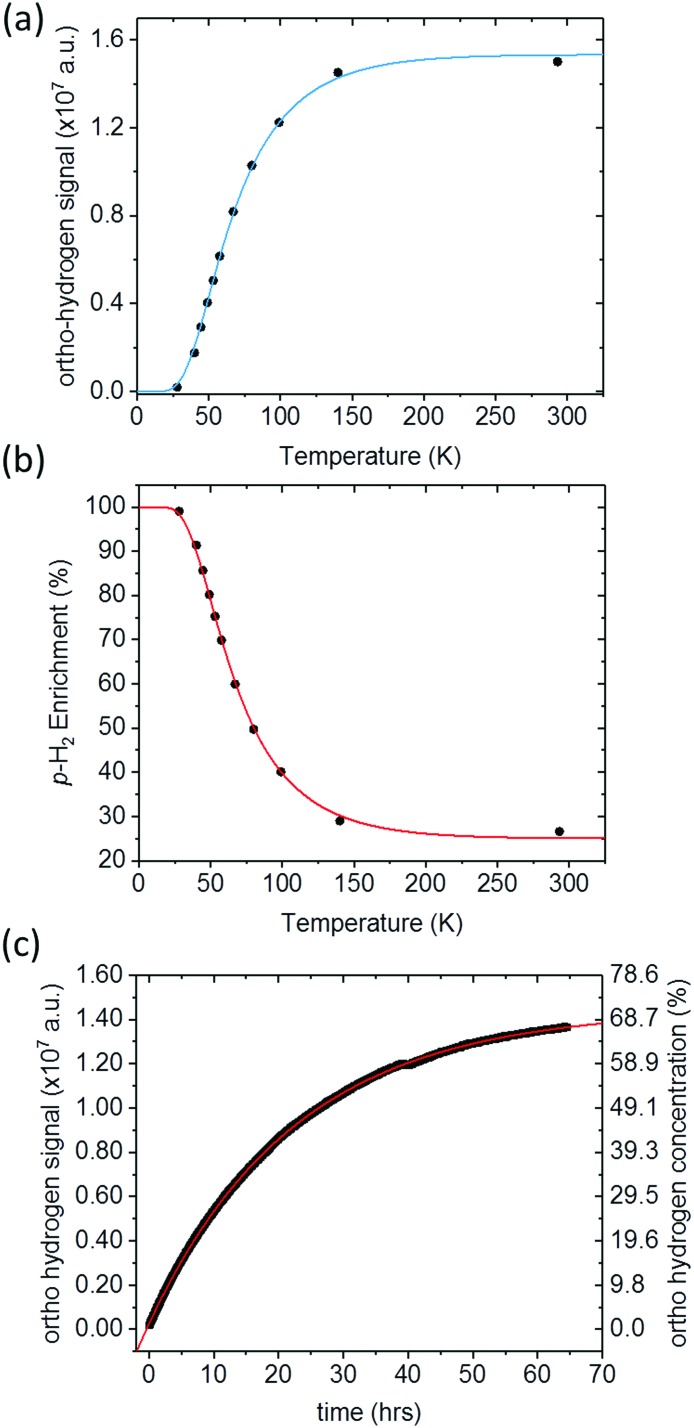
(a) ^1^H NMR signal response level of *p*-H_2_-enriched H_2_ gas dissolved in toluene (black circles), for conversion temperatures ranging from 28 K to 293 K. A fit to eqn (5) (blue line), gives a value of *θ*_R_ = (87.8 ± 0.7) K and *S*_max_ = (2.04 ± 0.02) × 10^7^, (b) the corresponding *p*-H_2_ enrichment calculated from eqn (6), where the red line was calculated using the fitted value of *θ*_R_. (c) ^1^H NMR *o*-H_2_ signal intensity monitored over 64 hours. At *t* = 0, hydrogen gas enriched to >99% *p*-H_2_ at a conversion temperature of 28 K was dissolved in toluene-*d*_8_ in an NMR tube and placed inside the NMR spectrometer. A fit to an exponential function to equilibrium (red line) reveals a time constant for the conversion to *o*-H_2_ in this case of 22.62 ± 0.02 hours.

Fig. 3a presents the ^1^H NMR response for *p*-H_2_-enriched H_2_ gas dissolved in toluene-*d*_8_ as a function of conversion temperature. The solid line is a fit to eqn (5), where the rotational constant was found to be *θ*_R_ = 87.8 ± 0.7 K. This is in excellent agreement with the value calculated previously from eqn (4) (87.57 K),^35^ as well as other experimentally determined values from the literature: *θ*_R_ = 87.6 K,^50^*θ*_R_ = 84.837 K,^36^ and *θ*_R_ = 85.3 K.^51^ Using the fitted value for the rotational constant, a plot of *p*-H_2_ enrichment as a function of conversion temperature can be produced from eqn (6), as demonstrated in Fig. 3b.

The liquid-state NMR approach that we used to quantify the *o*-H_2_^1^H NMR response assumes that an equal concentration of H_2_ is dissolved at each step of the experiment and that the conversion between *p*-H_2_ and *o*-H_2_ in solution is much slower than the timescale of our measurements. The validity of these assumptions is supported by the quality of the fits to the theoretical predictions in Fig. 3a and b. This also suggests that the generator is very efficient at cooling and has sufficient catalyst surface area to reach equilibrium before the gas has left the cooling block. Further confirmation was obtained by measuring the liquid-state ^1^H NMR response for H_2_ gas, enriched at 28 K and dissolved in toluene-*d*_8_, as a function of time inside the NMR spectrometer. The time-dependent ^1^H NMR response in Fig. 3c indicates a characteristic *para*-to-*ortho* exponential conversion time constant in solution of the order of 23 hours, which is comparable to conversion times observed previously in the gas phase^40^ and liquid phase.^41^ This is orders of magnitude slower than the tens of seconds required for each experimental measurement presented in Fig. 3a and b. The fit to the data in Fig. 3c shows that the signal tends to a value of (1.446 ± 0.001) × 10^7^ which is comparable to the intensity for H_2_ at room temperature of (1.501 ± 0.001) × 10^7^, which implies that the concentration of H_2_ in solution is not significantly changing throughout the experiment.

### Signal amplification by reversible exchange (SABRE)

**Fig. 4:**
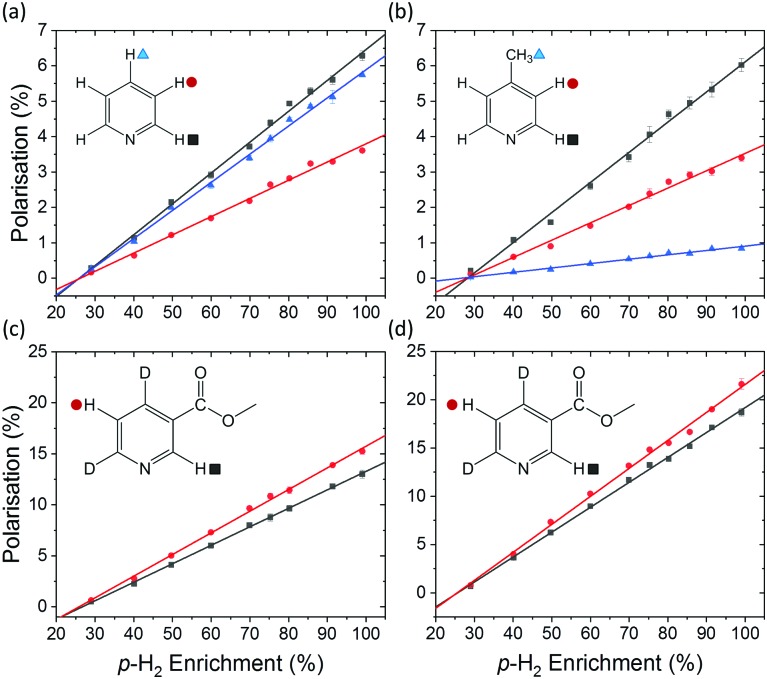
SABRE polarisation level as a function of *p*-H_2_ enrichment for (a) R_2_ = pyridine and R_1_ = IMes, (b) R_2_ = 4-methylpyridine and R_1_ = IMes, (c) R_2_ = methyl-4,6-*d*_2_-nicotinate and R_1_ = IMes, and (d) R_2_ = methyl-4,6-*d*_2_-nicotinate and R_1_ = *d*_22_-IMes. All spectra were acquired in methanol-*d*_4_ using a 400 MHz Bruker NMR spectrometer at 298 K. The data points and error bars represent the average and ± one standard deviation of five repeat measurements, respectively. The point at 99% *p*-H_2_ enrichment in (d) was calculated from SABRE-enhanced and thermally polarised ^1^H NMR spectra that were acquired with a de-tuned probe to avoid radiation damping effects.

A series of SABRE hyperpolarisation measurements were recorded using H_2_ gas containing known levels of *p*-H_2_ enrichment, calculated from the conversion temperature using the calibration curve in Fig. 3b. Fig. 4 presents the relationship between the level of *p*-H_2_ enrichment, *n*_*p*_, and the observed polarisation levels for the individual resonances of the target molecule, *P*_S_. It can be readily observed that the polarisation of the target molecule is linearly dependent on the percentage of *p*-H_2_ for all resonances. This effect is robust across different target molecules (see Fig. 4a–c) and for different SABRE catalysts (see Fig. 4c and d) and is consistent with the analytical model of SABRE hyperpolarisation developed by Barskiy *et al.*^52^

The slope of each linear correlation is a measure of the efficiency of the transfer of the latent polarisation of the *para*-enriched H_2_ to the target molecule. In order to quantify this, we define a SABRE efficiency parameter, *E*, as the ratio of the observed SABRE polarisation *P*_S_ to the polarisation of the *para*-enhanced H_2_ for a given level of *p*-H_2_ enrichment, *P*_H_2__.9
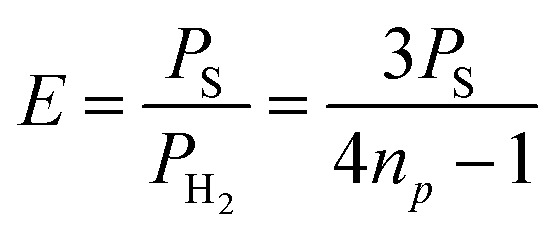
Rearranging we obtain a linear relationship between *P*_S_ and *n*_*p*_ with a slope of 4*E*/3.10
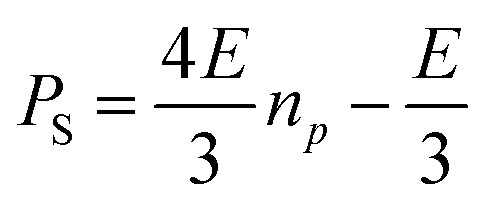
Therefore, the efficiency parameter can be extracted directly from the slope of each linear correlation in Fig. 4. We note that a similar approach has been used previously to quantify the efficiency of polarisation transfer when *p*-H_2_ is used in a hydrogenation reaction (*i.e.* hydrogenative PHIP).^53^

The SABRE efficiency parameters for the different substrate and catalyst systems in Fig. 4 are presented in Table 1. These can be used to directly compare the efficacy of SABRE hyperpolarisation for the different systems. First, we note that the efficiency of SABRE is not uniform within each molecule. This well-known feature of SABRE must be taken into account when developing a quantitative model for SABRE.^54^ However, the non-uniform distribution of polarisation is reproducible under fixed experimental conditions and so is unlikely to be a fundamental barrier to quantitative models based on SABRE.^54^,^55^

**Table 1:** SABRE efficiency values for the indicated resonances of the specified analytes as determined by eqn (9) and the lines of best fit in Fig. 4–6

Analyte (R_2_)	Catalyst NHC (R_1_)	SABRE-Relay carrier	NMR detection field *T* (MHz)	SABRE efficiency (*E*) (%)
Pyridine	IMes	N/A	9.4 (400)	2,6-H (*ortho*) 6.5 ± 0.1
				3,5-H (*meta*) 3.9 ± 0.1
				4-H (*para*) 6.0 ± 0.1

4-Methylpyridine	IMes	N/A	9.4 (400)	2,6-H (*ortho*) 6.4 ± 0.2
				3,5-H (*meta*) 3.7 ± 0.1
				4-CH_3_ (*methyl*) 0.93 ± 0.04

Methyl-4,6-*d*_2_-nicotinate	IMes	N/A	9.4 (400)	2-H (*ortho*) 13.6 ± 0.1
				5-H (*meta*) 15.9 ± 0.3

Methyl-4,6-*d*_2_-nicotinate	*d*_22_-IMes	N/A	9.4 (400)	2-H (*ortho*) 19.4 ± 0.3
				5-H (*meta*) 21.7 ± 0.5

Methyl-4,6-*d*_2_-nicotinate	IMes	N/A	1.0 (43)	2-H (*ortho*) 12.3 ± 0.2
				5-H (*meta*) 15.9 ± 0.2

1-Propanol	IMes	NH_3_	9.4 (400)	1-OH 0.57 ± 0.03
				1-CH_2_ 0.95 ± 0.05
				2-CH_2_ 0.57 ± 0.03
				3-CH_3_ 0.73 ± 0.03

Fig. 4c and d illustrate the benefits of selective deuteration of the target molecule. This is beneficial because it concentrates the available polarisation on fewer sites and it increases the relaxation times of the remaining protons.^45^ Finally, the use of a partially deuterated SABRE catalyst further improves the efficiency of the polarisation transfer.^56^ It is postulated that this is due to the combined effect of increasing the NMR relaxation times of the target molecules bound to the catalyst and limiting the distribution of *p*-H_2_-derived polarisation to the non-exchanging ligands. Thus there is a more efficient transfer to the target molecules bound *trans* to the hydrides in the active complex (see Fig. 1).^30^

In all of the examples presented in Fig. 4, a consistent experimental procedure was used. However, this method can also be used to compare the efficacy of different experimental implementations. For example, this approach could also be used to explore the efficiency of automated SABRE approaches, where *p*-H_2_ is bubbled through the solution and transferred to the NMR spectrometer for detection either manually^32^ or under flow.^57^,^58^

### NMR detection at 1 T

With the introduction of low-cost, homogeneous permanent magnet benchtop spectrometers, high quality sub-ppb spectra can be acquired, thus increasing the utility of benchtop NMR for a broad range of applications.^59^ It has been shown that the inherent sensitivity limit of low-field NMR can be overcome using various hyperpolarisation methods at fields from around 1 T down to ultra-low field.^60^–^62^

**Fig. 5:**
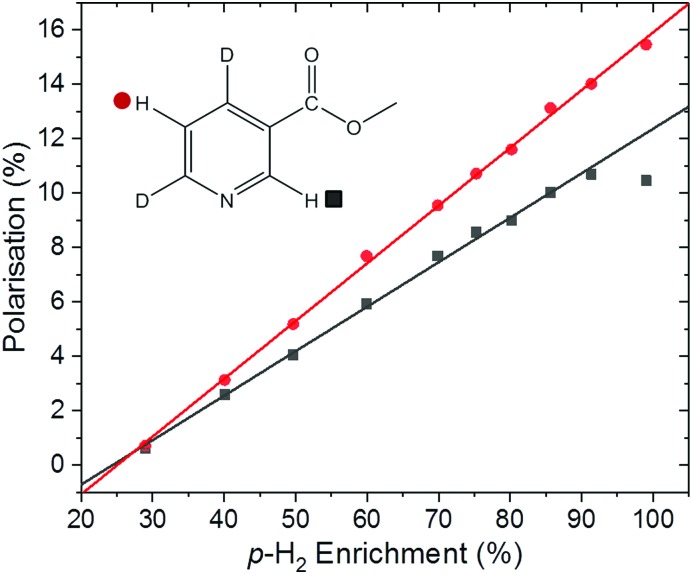
Methyl-4,6-*d*_2_-nicotinate hyperpolarisation level under SABRE as a function of *p*-H_2_ enrichment. Spectra were acquired on a 1 T benchtop NMR spectrometer (Magritek Spinsolve Carbon) and used the [IrCl(COD)(IMes)] pre-catalyst in methanol-*d*_4_. The final point at 99% *p*-H_2_ enrichment was acquired using a 30° pulse for both the SABRE experiment and corresponding thermal reference. The 2-H (*ortho*) position proton resonance still retained some antiphase character and gives a slightly reduced overall polarisation level as a consequence; it is omitted from the linear fit.

SABRE hyperpolarisation is generated outside of the NMR spectrometer in a relatively weak polarisation transfer field (typically 0–10 mT). Therefore, the polarisation level is expected to be independent of the magnetic field of the NMR spectrometer used for signal detection. Consequently, if all other experimental conditions (*e.g.* PTF, polarisation transfer time, and sample transfer time) are kept constant, we expect to measure the same SABRE efficiency response at different detection fields. To test this, the dependence of the SABRE polarisation of methyl-4,6-*d*_2_-nicotinate on the *p*-H_2_ enrichment was measured using a 1 T (43 MHz) benchtop NMR spectrometer for signal detection. The results in Fig. 5 illustrate that, as in the high-field case, a linear trend is found between the observed SABRE polarisation and the *p*-H_2_ enrichment. Furthermore, the calculated efficiency parameters for the two resonances of methyl-4,6-*d*_2_-nicotinate (12.3% and 15.9%) are in good agreement with those measured using a 400 MHz spectrometer for detection (13.6% and 15.9%).

It is important to note here an issue with the quantification of SABRE hyperpolarisation that we have frequently encountered for highly polarised samples. Under highly efficient SABRE conditions and when using high levels of *p*-H_2_ enrichment, peak broadening and anti-phase peak character is often observed in SABRE-enhanced ^1^H NMR spectra. We believe this to be a result of radiation damping. For the experiments in Fig. 4d, carried out on a 400 MHz NMR spectrometer, the spectra acquired at the highest level of *p*-H_2_ enrichment gave broad peaks with anti-phase character. However, narrow pure emission peaks were recovered by de-tuning the probe. This was only necessary for the final point in Fig. 4d corresponding to 99% *p*-H_2_. The resultant polarisation level, calculated using a reference measurement acquired with the de-tuned probe, followed the linear trend observed at lower *p*-H_2_ enrichment levels (Fig. 4d). In the benchtop NMR case (Fig. 5), the same solution could not be applied because the spectrometer cannot be de-tuned. Using a reduced tip-angle of 30°, a SABRE hyperpolarised spectrum was acquired which resulted in a narrow, pure emissive peak for the H-5 proton (red points in Fig. 5) with a corresponding polarisation level (calculated using an appropriately acquired reference spectrum) consistent with the linear trend. However, the H-2 resonance (black points in Fig. 5) retained anti-phase character. This gives an under-estimation of the polarisation level for the H-2 resonance (black squares) at the highest *p*-H_2_ enrichment level in Fig. 5, and so this point has been excluded from the linear fit. More details and example spectra are presented in the supporting information document.

Given the linear relationship between analyte polarisation and *p*-H_2_ enrichment level illustrated herein, we find that a more general solution to this issue for quantitative optimisation of highly efficient SABRE experiments is to use a lower level of *p*-H_2_ enrichment. If the level of *p*-H_2_ enrichment is known, a straightforward calculation of the efficiency parameter (eqn (9)) allows for extrapolation to the maximum achievable polarisation level at 100% *p*-H_2_ enrichment.

### SABRE-Relay

**Fig. 6:**
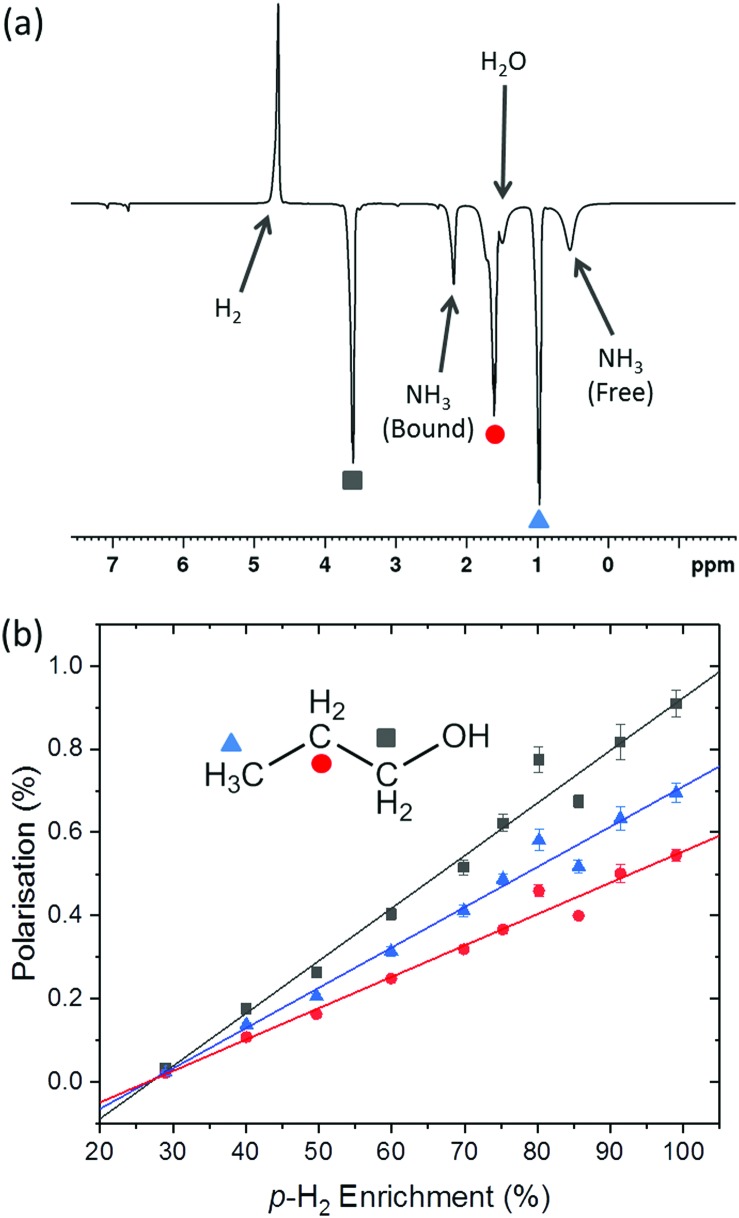
(a) An example SABRE-Relay hyperpolarised ^1^H NMR spectrum of 1-propanol. Data was acquired in a single scan on a 400 MHz Bruker NMR spectrometer at 298 K. (b) 1-Propanol SABRE-Relay hyperpolarisation level as a function of *p*-H_2_ enrichment when ammonia is the polarisation carrier and [IrCl(COD)(IMes)] the SABRE pre-catalyst in DCM-*d*_2_.

Thus far we have considered the effect of *p*-H_2_ concentration on the SABRE technique. However, it is also of interest to investigate the more recent SABRE-Relay method.^38^ In SABRE-Relay a carrier molecule is hyperpolarised through the standard SABRE mechanism. The polarisation of this carrier is then transferred to the target analyte through a subsequent reversible exchange reaction, such as proton exchange.^38^ Thus the target substrate molecule becomes hyperpolarised without interacting directly with the SABRE catalyst. In the example presented in Fig. 6, NH_3_ is used as the carrier and the target molecule is 1-propanol. The solvent, DCM-*d*_2_, is chosen as it has no exchangeable protons. However, if the solvent is not completely dry, any residual water present can participate in the proton exchange process and also become hyperpolarised.

Consider the SABRE-Relay hyperpolarised ^1^H NMR spectrum in Fig. 6a. The single absorption peak is assigned to hyperpolarised *o*-H_2_, while the emissive peaks are species in solution that have been hyperpolarised by SABRE (ammonia) or SABRE-Relay (1-propanol and water). We observe two resonances for NH_3_ that correspond to carrier molecules bound to the catalyst (2.19 ppm) and in free solution (0.51 ppm). In addition, we observe two well-resolved hyperpolarised peaks of 1-propanol: 1-CH_2_ (black square, 3.61 ppm) and 3-CH_3_ (blue triangle, 0.97 ppm), and one overlapping peak containing contributions from the 1-OH and 2-CH_2_ (red circle, 1.31–1.91 ppm). This set of overlapping peaks also contains a contribution from hyperpolarised water at 1.51 ppm. Using the same approach as for SABRE introduced above, we can evaluate the effect of *p*-H_2_ enrichment on the observed polarisation level of these three resonances, with the understanding that the third resonance contains the combined effects of 1-OH and 2-CH_2_ of 1-propanol as well as the residual water in the solvent. The results of this study, shown in Fig. 6b, indicate that, as in the SABRE case, the relationship between *p*-H_2_ enrichment and SABRE-Relay polarisation is linear. The corresponding SABRE-Relay efficiencies, calculated using eqn (9) (see Table 1) are lower than for the analytes polarised directly by SABRE but are nonetheless significant (maximum of 0.95% polarisation). As with the SABRE case, we find that the highest polarisation efficiency is observed for the proton resonance closest to the source of the hyperpolarisation (the exchanging 1-OH proton). Interestingly, the methyl group appears to have a higher efficiency than the 2-CH_2_, which is closer to the source of hyperpolarisation. However, this could simply be an artefact of the peak overlap, which acts to reduce the apparent hyperpolarisation of 2-CH_2_ due to the lower polarisation efficiencies of the water and the rapidly exchanging 1-OH proton. SABRE-Relay efficiency is dependent on the concentration of the carrier (ammonia in this case). Measurements of the concentration of ammonia in the SABRE-Relay samples using high-field ^1^H NMR showed some variability in the concentration of ammonia across the ten samples (standard deviation of 3.5%). This variability could account for the deviation from the linear relationship observed at 80% *p*-H_2_ enrichment in Fig. 6. Further details are provided in the supporting information document.

## Conclusions

In this work we have explored the polarisation transfer efficiency in SABRE and SABRE-Relay enhanced NMR spectroscopy for a range of experimental conditions. This was achieved by monitoring the observed hyperpolarisation of a target analyte as a function of the level of *para*-enrichment of the H_2_ gas used. A linear relationship was found over the range of enrichment levels spanning 29 to 99% for different target analytes, polarisation transfer catalysts, NMR detection fields, and for both the SABRE and SABRE-Relay polarisation transfer mechanisms. The reproducibility and universality of this linear relationship suggests that the distribution of polarisation within a SABRE hyperpolarised system is in a steady-state and can provide a highly reproducible NMR response for future quantitative applications.

The gradient of each linear correlation was related to a standard theoretical model of *p*-H_2_ to define an efficiency parameter, *E*, that quantifies the fraction of the available *p*-H_2_ polarisation that is transferred to the target analyte under the given experimental conditions. It was shown that the SABRE efficiency is independent of the NMR detection field, as expected, and is comparable for analytes with similar chemical properties such as pyridine and 4-methylpyridine. High SABRE efficiencies of up to 21.7% were observed for a selectively deuterated substrate, methyl-4,6-*d*_2_-nicotinate, in conjunction with a highly efficient SABRE catalyst [Ir(H)_2_(methyl-4,6-*d*_2_-nicotinate)_3_(*d*_22_-IMes)].^45^ This result supports previous conclusions regarding the benefits of selective deuteration for improved ^1^H SABRE hyperpolarisation through the combined effects of extending ^1^H hyperpolarisation lifetimes and the concentration of the available hyperpolarisation across fewer ^1^H resonances.^45^

The hyperpolarisation of 1-propanol *via* SABRE-Relay exhibited a polarisation transfer efficiency of up to 0.95%. While significant, this transfer efficiency is an order of magnitude lower than that for direct SABRE hyperpolarisation. Given that SABRE-Relay is a relatively new development, this suggests that there is significant scope for further optimisation. For example, the polarisation transfer conditions have been optimised for the first stage of the SABRE-Relay process (transfer to the carrier) and not the second stage: the relay of polarisation to the target analyte.^38^

The efficiency measurements were made possible by using a *p*-H_2_ generator that can access interconversion temperatures ranging from 28 K to 140 K, yielding *p*-H_2_ enrichment levels between around 99% and 28%. The quantitative relationship between the conversion temperature and the level of *p*-H_2_ enrichment was verified experimentally using liquid-state ^1^H NMR and the standard theoretical description of *p*-H_2_ and *o*-H_2_. We have shown that the *p*-H_2_ concentration can be accurately and efficiently measured using NMR spectroscopy in a single scan if the hydrogen gas is first dissolved into solution. This provides a sensitivity advantage over the more established method of gas-phase NMR detection, where 256 scans were required.^40^ The amount of dissolved H_2_ gas in solution was found to be stable over a 64 hour measurement window, with conversion from the *p*-H_2_ back to equilibrium with a relaxation time of ∼23 hours. This suggests that the levels of *p*-H_2_ enrichment were constant over the timescales of our SABRE measurements (order of minutes).

Although a clear sensitivity advantage is gained by using the highest available level of *p*-H_2_ enrichment, low-temperature generators like the one detailed herein come with significant costs for both the initial purchase and maintenance. Using the efficiency parameter defined herein, the sensitivity implications of the use of the lower levels of *p*-H_2_ enrichment associated with lower-cost generators can be quantified, and so an appropriate cost-to-sensitivity trade off can be determined for a given application of SABRE-enhanced NMR or MRI.

## Data access statement

All experimental NMR data reported in this work is available *via* Research Data York at ; http://dx.doi.org/10.15124/80f03d83-971b-4bc0-a4ef-81a46cfbdcfe.

## Conflicts of interest

There are no conflicts to declare.

## Supplementary Material

Supplementary information
