# Supplementary material for: Quantification of hyperpolarisation efficiency in SABRE and SABRE-Relay enhanced NMR spectroscopy
Source: Phys Chem Chem Phys. 2018 Oct 10;20(41):26362–71. doi: 10.1039/c8cp05473h (PMC6202922; doi:10.1039/c8cp05473h)
Supplement: Supplementary file 1 [file CP-020-C8CP05473H-s001.pdf]

Electronic Supplementary Material (ESI) for Physical Chemistry Chemical Physics.
This journal is © the Owner Societies 2018

Quantification of hyperpolarisation efficiency in SABRE and SABRE-Relay enhanced NMR spectroscopy

Peter M. Richardson,[a] Richard O. John,[a] Andrew J. Parrott,[b] Peter J. Rayner,[a] Wissam Iali,[a] Alison Nordon,[b] Meghan E. Halse*[a] and Simon B. Duckett*[a]

[a]*Centre for Hyperpolarisation in Magnetic Resonance, Department of Chemistry, University of York, UK*

[b]*Department of Pure and Applied Chemistry, University of Strathclyde, Glasgow, UK*

Contents

Para-hydrogen Generator 3

Radiation Damping at 9.4 T 4

Signal Saturation at 1 T 6

SABRE-Relay Sample Composition 7

Para-hydrogen Generator

The *para*-hydrogen generator used in this work is a bespoke design to allow fast delivery of high purity *para*-hydrogen. The cold head, which is a closed-loop helium refrigeration device allows temperatures down to 7 K, has an attached copper block. This coupled with a heater controlled by a feedback loop provides access to a range of interconversion temperatures. The H_2 gas flows through the copper block, which contains a paramagnetic catalyst to provide efficient conversion to the *para* form. Once the *p*-H_2 is formed it can be easily added to an NMR tube via an adaptor. Once the *p*-H_2 has been used it can be evacuated from the headspace of the NMR tube using the same system, thus allowing fresh *p*-H_2 to be added. The schematic in Figure S1 highlights the operation of the *p*-H_2 rig, where the circles represent valves in the system and the boxes represent the various components used.

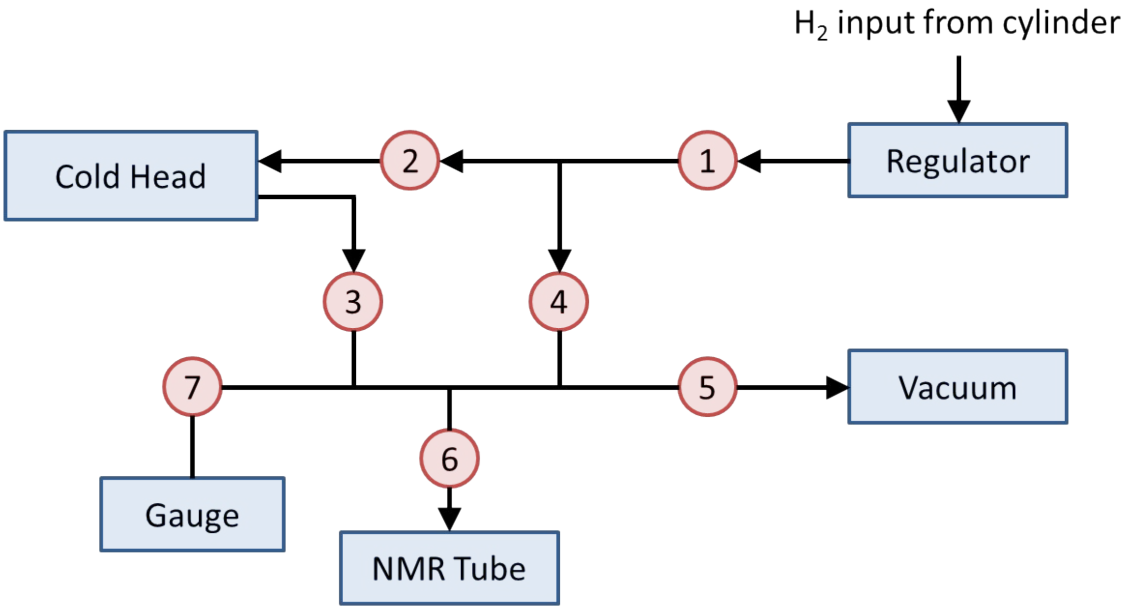

Figure S1 – Schematic of the para-hydrogen generator used in this research. The circles and rectangles denote valves and components, respectively.

H_2 is supplied via an external cylinder which contains a regulator set to 4 bar absolute pressure. Valve 1 is present to isolate and purge the whole system in the case of any blockages. Valve 2 allows the cold head block to be isolated during the purging procedure, which involves cycles of flushing H_2 through the cold head block and then evacuating before reducing the temperature. Under standard operation valves 1 and 2 remain open, valves 3 and 4 remaining closed and the system is under vacuum (valve 5 open). Valve 6 can be used when an NMR tube fitted with a Young's valve is in place. Before opening the Young's valve, the NMR tube is attached via the adapter and valve 6 is opened to allow the line to be evacuated to the top of the tube. Before filling with *p*-H_2, it is standard to flush and purge the system with H_2 directly from the cylinder by alternating between opening and closing valves 4 and 5. Once the lines are purged, *p*-H_2 can be added to the NMR tube by opening valve 3 and subsequently opening the top of the NMR tube via the Young's valve. In the case of repeat measurements with the same sample, the headspace of the NMR tube is first evacuated by closing valve 5 and opening the Young's valve to expose the headspace of the NMR tube to the residual vacuum and then fresh *p*-H_2 is added.

Radiation Damping at 9.4 T

The methyl-4,6-d_2-nicotinate substrate with the deuterated catalyst [IrCl(COD)(d_{22}-IMes)] was the most efficient SABRE system presented here. At the highest purity of p-H_2 (99 %) the polarisation level reached around 22 % for a substrate concentration of 26 mM. Under these conditions, an antiphase signal was observed for all repeats of the SABRE hyperpolarisation experiment. We hypothesise that this behavior is due to radiation damping because it was only observed for the system with the largest raw NMR signal intensity. An example spectrum obtained by performing SABRE on a solution of 26 mM methyl-4,6-d_2-nicotinate with 5.2 mM of the deuterated catalyst [IrCl(COD)(d_{22}-IMes)] in methanol-d_4 with 10 s of shaking in a 63 G magnetic field is shown in Figure S2. It can be readily observed that the signal is significantly antiphase in character.

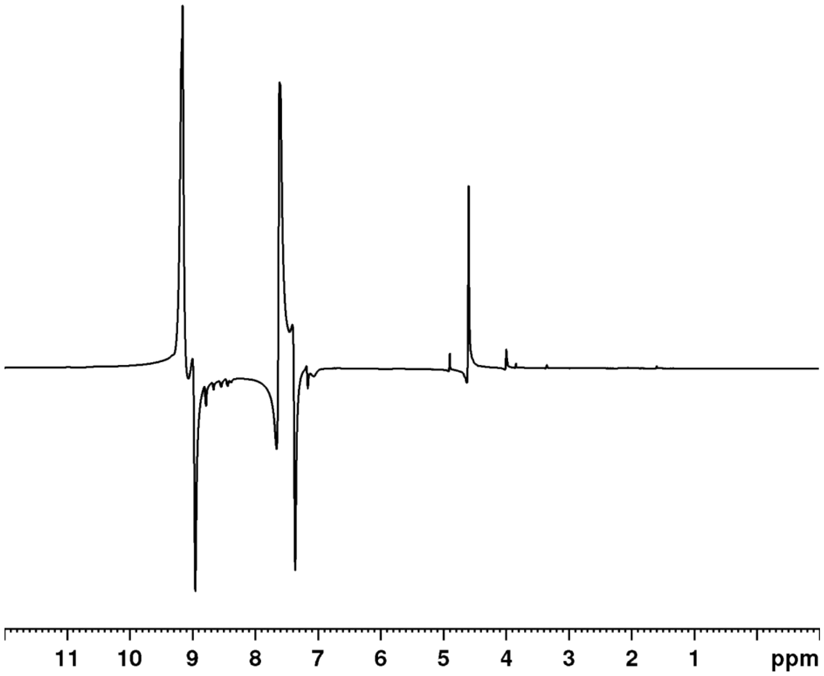

Figure S2 – SABRE spectrum for sample containing 26 mM methyl-4,6-d_2-nicotinate with 5.2 mM of the deuterated catalyst [IrCl(COD)(d_{22}-IMes)] in methanol-d_4 with shaking with 4 bar para-hydrogen for 10 s in a 63 G shaker.

In order to circumvent the radiation damping effect, the probe was detuned away from the resonant frequency and the SABRE hyperpolarisation was re-measured with the same conditions as in Figure S2. The resulting spectrum, shown in Figure S3, shows pure emission SABRE peaks. In order to calculate an accurate enhancement factor for this system, a thermally-polarised reference spectrum was acquired with the same tuning. The resulting polarisation value, displayed in Figure 3d of the main text, is in good agreement with the linear trend at lower p-H_2 enrichment levels.

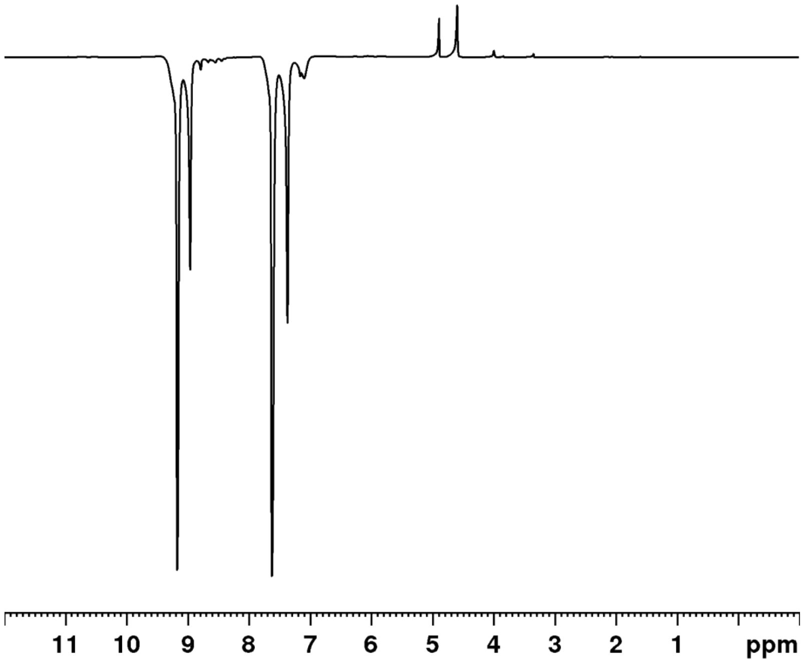

Figure S3 – Detuned SABRE spectrum for sample containing 26 mM methyl-4,6-d_2-nicotinate with 5.2 mM of the deuterated catalyst [IrCl(COD)(d_{22}-IMes)] in methanol-d_4 with shaking with 4 bar para-hydrogen for 10 s in a 63 G shaker.

Signal Saturation at 1 T

In Figure 5 of the main text we show the SABRE polarisation for a range of *p*-H_2 concentrations as measured on a 1 T Magritek Spinsolve Carbon benchtop NMR spectrometer. The sample used contained 26 mM of the most efficient substrate presented here methyl-4,6-d_2-nicotinate with 5.2 mM [IrCl(COD)(IMes)] catalyst in methanol-d_4. As shown in Figure 5 of the main text the relationship of polarisation and *p*-H_2 enrichment was linear, as expected and the slopes of the linear correlations are in good agreement with those detected at high field. However, at the highest enrichment of 99 % the polarisation of the substrate appeared to reach a plateau. The benchtop spectrometer cannot be detuned and therefore to test the origin of this effect, we acquired a SABRE-enhanced NMR spectrum with 30° excitation pulse to reduce the level of signal induced in the receiver. The resulting spectrum is shown in Figure S4. While the line-shapes of both resonances are not fully emissive, when compared to a thermally polarised spectrum acquired using the same 30 excitation pulse, the enhancement factor of the meta resonance (7.46 ppm) is consistent with the linear trend in Figure 5. The ortho resonance (9.04 ppm) peak enhancement factor lies below the expected linear trend due to the antiphase character. The observation that a smaller pulse angle, which yields a proportionally lower level of detectable magnetisation, restores the meta resonance to the expected linear trend suggests that the observed signal plateau under conditions of high SABRE efficiency is a function of the benchtop NMR detector and not the underlying polarisation of the sample.

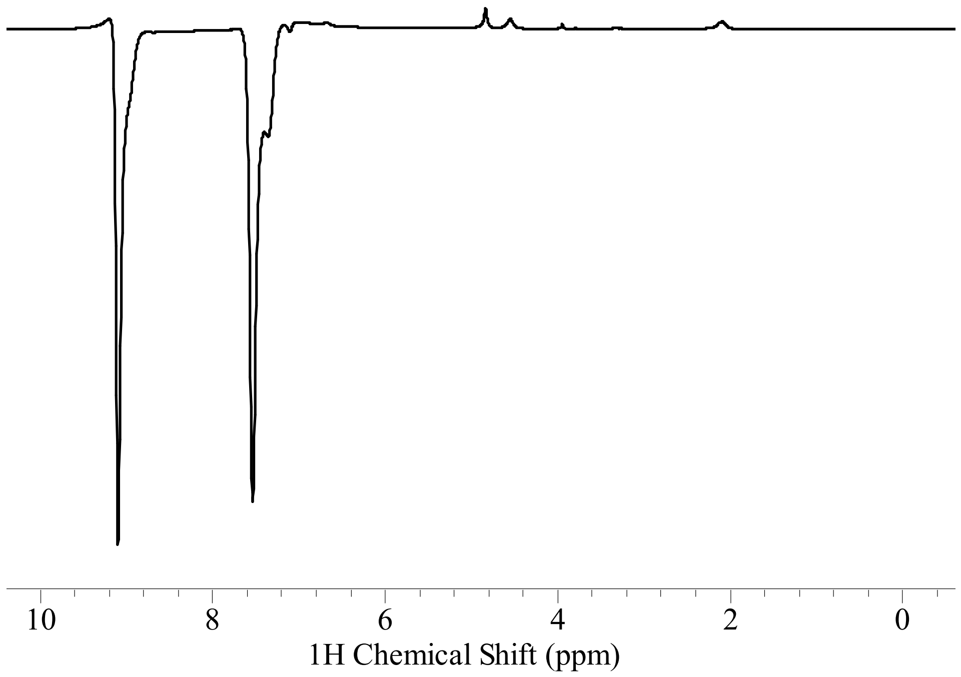

Figure S4 – SABRE hyperpolarised ^{1}H NMR spectrum of 26 mM methyl-4,6-d2-nicotinate with 5.2 mM [IrCl(COD)(IMes)] catalyst in methanol-d_4. This spectrum was acquired with a 30° pulse to avoid signal saturation on the benchtop NMR spectrometer.

SABRE-Relay Sample Composition

The SABRE-Relay samples require a carrier amine to successfully transfer polarisation to the target molecule. Here we exemplified the use of ammonia as the carrier agent; this amine has been shown to work very efficiently with SABRE-Relay and therefore is a desirable carrier to use here. However, for this study it was important that all samples contained the same constituents. In order to achieve this, a stock solution was created containing the target substrate (1-propanol), solvent (DCM-d_2) and catalyst ([IrCl(COD)(IMes)]). This was placed inside a 10 mm Young's NMR tube; subsequently the tube was degassed using a freeze-pump-thaw procedure using liquid nitrogen. Ammonia gas was then added to the tube and vigorously shaken to dissolve the ammonia in solution. The solution was then distributed to ten different 5 mm Young's valve NMR tubes for analysis with SABRE-Relay.

Before activation of the catalyst the amount of ammonia is each sample was determined via ^{1}H NMR spectroscopy. This was achieved by taking NMR spectra of each sample and comparing the integral of a known catalyst peak and to the integral of the ammonia in the spectrum (see Figure S5 for example spectrum). The unactivated catalyst has the form [IrCl(COD)(IMes)] where the COD is 1,5-cyclooctadiene which binds through the two CH_2 resonances. These resonances occur at 3.03 ppm and 4.07 ppm and are inequivalent due to the ligation to the metal centre. This allowed the determination of an equivalence of ammonia with respect to the iridium catalyst by using the equation S1.

$$NH_3\text{:}[IrCl(COD)(IMes)] = \frac{2I_{NH_3}}{3I_{COD}}\text{:}1$$

The factor of 2/3 arises from the COD and the ammonia peaks corresponding to two and three protons, respectively. In the results for all samples are presented in Table S1.

Table S1 – Integrals of catalyst peak and ammonia peak to ascertain the amount of ammonia with respect to the catalyst. It was found that on average around 8:1 ammonia: catalyst was present throughout the samples. All were measured at 298 K on a 400 MHz high-field spectrometer. The error on the ammonia equivalence is determined from error propagation from three repeat spectra.

Interconversion Temperature (K)	p-H2 (%)	Catalyst Peak (3.03 ppm, H2) Integral (a.u.)	Ammonia (0.5 ppm, H3) Integral (a.u.)	Ammonia equivalence
140.0	28.70	5536477.885	65984454.78	7.95 ± 0.2
99.0	39.85	5500229.427	65397109.67	7.93 ± 0.1
80.0	49.49	5500525.345	68391755.86	8.29 ± 0.06
67.0	59.78	5569683.705	68037500.67	8.14 ± 0.09
57.5	69.77	5459640.010	68074770.46	8.31 ± 0.08
53.0	75.17	5514827.773	64431539.17	7.79 ± 0.1
49.0	80.13	5676426.957	62605734.03	7.35 ± 0.08
44.5	85.60	5543391.013	66823028.23	8.04 ± 0.04
40.0	91.34	5612401.150	66731315.06	7.93 ± 0.2
28.0	99.04	5493599.723	66897166.28	8.12 ± 0.1
			average	7.98
			stdev	0.28

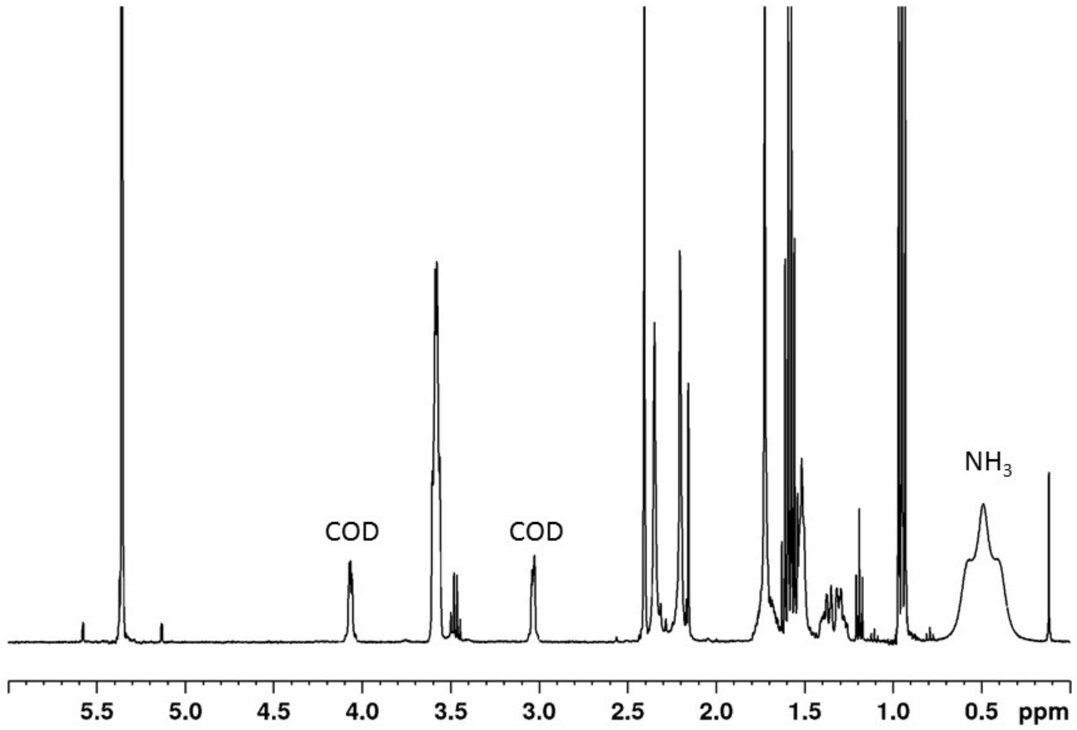

Figure S5 – Thermal spectrum for sample containing ammonia (0.5 ppm) with 5.2 mM of the catalyst [IrCl(COD)(IMes)] in DCM-d_2 before the addition of H_2 and therefore unactivated catalyst. The ratio of catalyst to ammonia can be determined by comparing the ammonia peak with a known resonance on the catalyst. The 1,5-cyclooctadiene has two CH_2 resonances which are inequivalent due to ligation on the catalyst. The resonances at 3.03 ppm and 4.07 ppm correspond to the two CH_2 resonances. Both resonances were found to give very comparable signals so the peak at 3.03 ppm was arbitrarily chosen for comparison. Using equation S1 the ratio of catalyst to ammonia can be readily determined from the NMR integrals.

For all but three samples the ratio of catalyst to substrate ratio stayed within one standard deviation away from the average. The samples which were used with the 80 K, 57.5 K and 49 K conversion temperatures were all outside one standard deviation. The 80 K and 57.5 K samples were 1.1 and 1.2 standard deviations away, which is only slightly outside the single standard deviation. However the sample which used the 49 K (80.13 % *p*-H_2) conversion temperature gave a ratio of 7.35 which is far from the average of 7.98, and lies 2.25 standard deviations away. The efficiency of SABRE-Relay is very sensitive to the concentration of the carrier. Therefore this difference in concentration could explain the result in Figure 6b of the main text, which shows a polarisation which is higher than expected from the linear fit for this sample.
